# Head-up tilt test induces T-wave alternans in long QT syndrome with KCNQ1 gene mutation: Case report CARE-compliant article

**DOI:** 10.1097/MD.0000000000019818

**Published:** 2020-05-15

**Authors:** Beiyin Gu, Tingliang Liu, Lei Yang, Haiyan Zhang, Yili Xin, Jian Wang

**Affiliations:** Department of Cardiology, Shanghai Children's Medical Center, Shanghai Jiaotong University School of Medicine, Shanghai, China.

**Keywords:** head-up tilt test, long QT syndrome, T-wave alternans

## Abstract

**Introduction:**

Long QT syndrome (LQTS) is a congenital disorder characterized by a prolongation of the QT interval on electrocardiograms (ECGs) and a propensity to ventricular tachyarrhythmias, which may lead to syncope, cardiac arrest, or sudden death. T-wave alternans (TWA) refers to the periodic beat-to-beat alternation of T-wave shape, polarity and amplitude on surface ECG during regular heart rhythm. In this report, a case of long QT syndrome with KCNQ1 gene mutation induced TWA in the head-up tilt test (HUTT), which has not been reported yet.

**Patient concerns:**

A 6-year-old boy presented with loss of consciousness twice, 5 months in duration. The boy's ECG showed prolonged QT interval (QTc = 600 ms, QTc = QT/RR^1/2^). During HUTT test, QT interval was significantly prolonged (QTc = 716 ms) based on macroscopic TWA.

**Diagnosis:**

The patient was diagnosed with 1. Long QT syndrome type 1(LQT1); 2. Vasovagal syncope (VVS)

**Interventions:**

Metoprolol 12.5 mg was given orally twice a day. The child was told avoid standing for a long time and strenuous exercises.

**Outcomes:**

There was no syncope or arrhythmia occurred during hospitalization and follow-up for 1 year.

**Conclusions:**

VVS may exist in patients with long QT syndrome. Increased sympathetic tone during the early stage of HUTT may induce macroscopic TWA in long QT syndrome with KCNQ1 gene mutation.

## 1 Introduction

Long QT syndrome (LQTS) is a congenital disorder characterized by a prolongation of the QT interval on electrocardiograms (ECGs) and a propensity to ventricular tachyarrhythmias, which may lead to syncope, cardiac arrest, or sudden death ^[1,2]^. T-wave alternans (TWA) refers to the periodic beat-to-beat alternation of T-wave shape, polarity and amplitude on surface ECG during regular heart rhythm ^[3,4]^. In this report, a case of long QT syndrome (LQTS) with KCNQ1 gene mutation induced TWA in the head-up tilt test (HUTT), which has not been reported yet.

## 2 Clinical data

A 6-year-old boy was admitted to our hospital with history of 2 episodes of loss of consciousness during the past 5 months. The symptom was the loss of consciousness without incontinence of urine or stool, and was improved after few minutes. The child had no fever, convulsions, cough, vomiting, diarrhea or loss of consciousness. The child was admitted for further diagnosis and treatment. His family history included normal parents, and his elder sister died suddenly in school at the age of 12, while the cause of death was unknown. There was no history of head trauma or drug ingestion. The child was clear-minded, and satisfactory mental reaction, moderate nutrition, clear breath sound in both lungs, and no rale were found; the heart rate (HR) was 58 times/min, being rhythmic and powerful; no obvious murmur was found as well.

**Figure 1:**
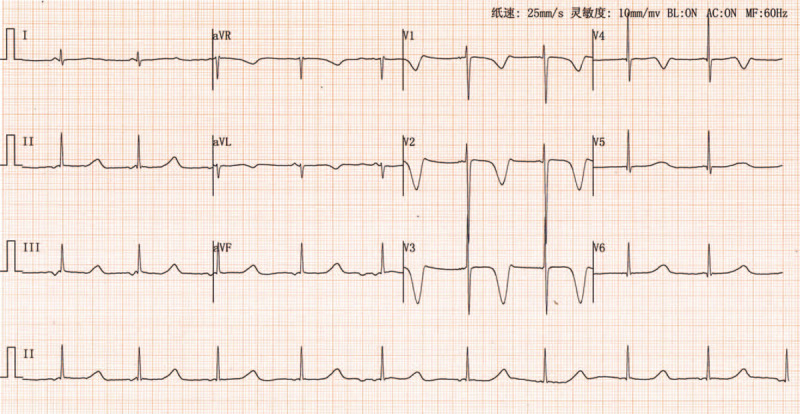
ECG shows arrhythmia at the atrioventricular junction (HR: 58 beats/min) and significantly prolonged QT interval (QTc = 600 ms). ECG = electrocardiogram.

**Figure 2:**
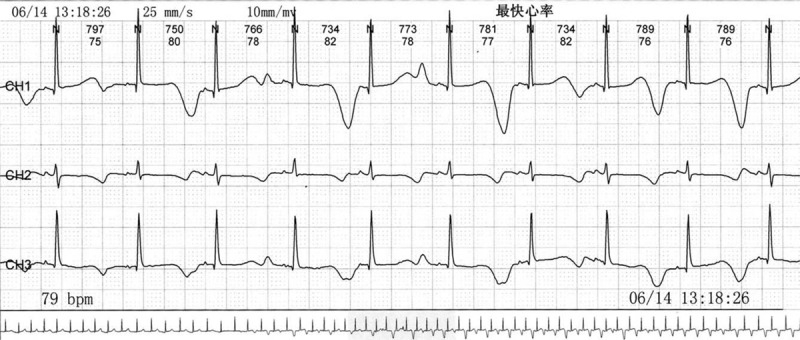
ECG in HUTT: sinus rhythm (HR: 79 beats/min) and a considerable prolonged QT interval (QTc = 716 ms) with significant TWA. ECG = electrocardiogram, HUTT=head-up tilt test.

Auxiliary examinations included measurement of the following parameters: blood routine: the number of white blood cells was 7.76 × 10^9^/L; the rate of lymphocyte was 39.4%; the rate of neutrophil was 51.3%; the level of hemoglobin was 135.0 g/L; and platelet count was 349∗10^9^/L. CK: 74U/L, CK-MB: 1.5ug/mL; the level of NT-ProBNP was 52 pg/mL; function of liver and kidney was normal; the level of fasting blood glucose was normal; normal sleep electroencephalogram was observed; 24-hours ambulatory electroencephalogram showed a normal level; cranial computed tomography scan showed no obvious abnormality; echocardiogram was normal; ECG showed prolonged QT interval (QTc = QT/RR^1/2^, QTc = 600 ms) (Fig. 1). Additionally, 24-hours ambulatory ECG showed no ventricular arrhythmia and prolonged QT interval (QTc) was 471-566 ms. Screening ECGs of the parents were normal. HUTT showed vasovagal syncope-vascular depression. 4 minutes after 60 degrees tilt and stood upright, the child felt abdominal pain accompanied by pale face, and then dizziness, without blurred vision or syncope; blood pressure and HR were 113/47 mm Hg and 79 times/min, respectively, and QT interval was significantly prolonged based on macroscopic TWA (Fig. 2). The symptoms were improved after lying flat DNA sequencing revealed “missense mutation” in KCNQ1 gene, c. 1022C>T. p. Ala341Val (heterozygosis). The patient's father was carrier of this mutation (heterozygosis), and his mother was carrier of the normal genotype.

Metoprolol 12.5 mg was given orally twice a day during hospitalization and discharge. During hospitalization, 1 g of sodium creatine phosphate was given intravenously twice a day for two weeks. After discharge, 1 g/10 mL of fructose sodium diphosphate oral solution was given orally twice a day for 2 weeks. The child was told avoid standing for a long time and strenuous exercises. There was no syncope or arrhythmia occurred during hospitalization and follow-up 1 year. It is noteworthy that the patient's family members signed informed consent forms before start of testing. The patient's parents have provided informed consent for publication of the case.

## 3 Discussion

It has been reported that TWA is observed in LQTS patients, that is mainly associated with malignant arrhythmias ^[5,6]^. In 1994, Rosenbaum et al reported that TWA can be a significant independent predictor of persistent tachyarrhythmia ^[7]^. The mechanism of TWA has not been fully elucidated, however, it is generally believed that there are 3 possible mechanisms: electrophysiological mechanism, ionic mechanism, and neurological mechanism ^[8]^.

Cruz et al studied 11 patients with LQTS and observed that TWA was closely associated with ventricular arrhythmia; this phenomenon was often common in emotional excitation or physical activity^[9]^. During the HUTT, significant TWA was induced, which may be related to the increase of sympathetic nerve tension caused by early stage of HUTT. Increased sympathetic nerve tension further prolongs the QT interval in the LQTS children, accompanied by TWA, with a potential risk of malignant ventricular arrhythmias ^[10,11,12]^.

KCNQ1 is the most common pathogenic gene of LQTS. In addition, KCNQ1 encodes Kv7.1 α-subunit and forms a slowly activated a delayed rectifier potassium current channel (Iks channel) with the accessory protein KCNE1. This potassium channel can be stimulated by β-adrenergic, and was up-regulated by cAMP-PKA-KCNQ1 pathway and AKAP-9-involved signaling transduction pathway ^[13]^. In sympathetic nerve activity, adrenergic stimulation increases outward current, counteracts the corresponding increase of calcium influx, and shortens the QT interval when HR increases. The mutated Iks current cannot effectively adapt to the stimulation of β-adrenaline to produce this up-regulation function, resulting in arrhythmia, that mat produce a proarrhythmic effect, making the affected individuals more prone to heart failure events, especially during exercise and emotional stimulation.

The mechanism of hemodynamic changes during vasovagal syncope, that is generally recognized as Bezold-Jarisch reflex ^[14]^. When venous congestion occurs in the lower extremities of patients with vasovagal syncope, the rapid decrease of left ventricular blood volume excites the Bezold-Jarisch reflex ring, and the reflexivity increases the sympathetic nerve tension, leading to a hypersystolic state of nearly complete ventricular emptying, thereby stimulating the C fiber mechanoreceptors in the posterior-inferior wall of left ventricle, enhancing the activity of the vagus nerve, as well as causing the decrease of peripheral vascular resistance and/or HR, loss of consciousness, and syncope attack in severe patients.

In our case, the HUTT was performed to determine the cause of syncope. Although the ECG showed that the QT interval was prolonged, however, there was no evidence that it was the cause of syncope. It was necessary to differentiate it from vasovagal syncope. The symptoms and hypotension were developed at the early stage of tilt. A diagnosis of LQT1 and VVS were made and the child was started on Metoprolol 12.5 mg bid orally. The child was also advised to avoid standing long and strenuous exercises. There was no syncope or arrhythmia occurred during treatment.

VVS may exist in patients with long QT syndrome, especially LQT1^[15]^. Increased sympathetic tone during the early stage of HUTT may induce macroscopic TWA in LQTS with KCNQ1 gene mutation. The possibility of inducing TWA by HUTT may be a proper method of risk stratification of prognosis in LQTS children, which involving a great clinical significance.

## Author contributions

**Conceptualization:** Beiyin Gu.

**Data curation:** Tingliang Liu, Jian Wang.

**Formal analysis:** Beiyin Gu.

**Investigation:** Beiyin Gu, Yili Xin.

**Methodology:** Tingliang Liu.

**Project administration:** Lei Yang.

**Resources:** Haiyan Zhang, Jian Wang.

**Supervision:** Yili Xin.

**Validation:** Lei Yang, Haiyan Zhang.

**Writing – original draft:** Beiyin Gu.

**Writing – review and editing:** Beiyin Gu, Tingliang Liu.
